# The incidence of drug-induced interstitial lung disease caused by epidermal growth factor receptor tyrosine kinase inhibitors or immune checkpoint inhibitors in patients with non-small cell lung cancer in presence and absence of vascular endothelial growth factor inhibitors: a systematic review

**DOI:** 10.3389/fonc.2024.1419256

**Published:** 2024-06-11

**Authors:** Yutaka Fujiwara, Kazuhiro Shimomura, Teppei Yamaguchi, Junichi Shimizu, Naohiro Watanabe, Reiko Matsuzawa, Kenta Murotani, Yoshitsugu Horio

**Affiliations:** ^1^ Department of Thoracic Oncology, Aichi Cancer Center, Nagoya, Japan; ^2^ Department of Pharmacy, Aichi Cancer Center, Nagoya, Japan; ^3^ Biostatistics Center, Kurume University, Kurume, Japan; ^4^ School of Medical Technology, Kurume University, Kurume, Japan

**Keywords:** epidermal growth factor receptor, immune checkpoint inhibitor, interstitial lung disease, non-small cell lung cancer, pneumonitis, tyrosine kinase inhibitor, vascular endothelial growth factor

## Abstract

Interstitial lung disease (ILD) or pneumonitis caused by epidermal growth factor receptor (EGFR) tyrosine kinase inhibitors (TKI) or immune checkpoint inhibitors (ICI) is a major concern in the treatment of non-small cell lung cancer (NSCLC). Whether the addition of vascular endothelial growth factor (VEGF) and VEGF receptor (VEGFR) inhibitors can reduce the incidence of drug-induced ILD remains unclear. We conducted a systematic review to assess the incidence of ILD induced by EGFR-TKIs or ICIs in the presence or absence of VEGF/VEGFR inhibitors in relevant randomized trials between January 2009 and October 2023. The primary outcome was the odds ratio for the incidence of ILD in all patients worldwide and Asians. Secondary outcomes were the odds ratios (ORs) of the incidence at grade-3 or higher ILD in all patients worldwide and Asians. We identified 13 randomized studies, one sub-analysis in the EGFR-TKI group, and three randomized studies in the ICI group. In the EGFR-TKI group, the OR of ILD incidence at any grade with VEGF/VEGFR inhibitors was 0.54 (95% CI, 0.32–0.90; p = 0.02), which represented a significantly lower incidence than that without VEGF/VEGFR inhibitors. Contrarily, the OR of ILD incidence at grade ≥ 3 with VEGF/VEGFR inhibitors was 1.00 (95% CI, 0.43–2.36; p = 0.99). In all subjects in the ICI group, the OR of ILD incidence at any grade with VEGF/VEGFR inhibitors was 0.78 (95% CI, 0.51–1.21; p = 0.27). The systematic review demonstrated that the addition of VEGF/VEGFR inhibitors could reduce the incidence of drug-induced ILD at any grade caused by EGFR-TKI in patients with NSCLC but could not reduce that at grade ≥ 3. The ILD induced by ICIs remains undetermined owing to the limited number of randomized trials for which ILD data are available.

**Systematic review registration:**

https://www.crd.york.ac.uk/PROSPERO/display_record.php?RecordID=409534, identifier CRD42023409534.

## Introduction

The discovery of molecular-targeted agents for driver gene alterations and immune checkpoint inhibitors (ICI) has led to a dramatic paradigm shift in therapeutic strategies for advanced non-small-cell lung cancer (NSCLC) (1–3). Currently, standard chemotherapy for advanced NSCLC is administered based on the molecular subtype of the tumor and the programmed death-ligand 1 (PD-L1) status in tumor cells. The most common druggable gene alteration in patients with NSCLC is epidermal growth factor receptor (EGFR) mutation, occurring in 30–50% of East Asians and 10–15% of Westerners (4). First- and second-generation EGFR tyrosine kinase inhibitors (TKIs) such as erlotinib and afatinib, have demonstrated impressive clinical activity and superiority over chemotherapy in patients with EGFR-mutated NSCLC. The third-generation EGFR TKI; osimertinib, potently and selectively inhibits both EGFR-TKI sensitizing and EGFR T790M resistance mutations. Randomized phase 3 studies of AURA3 and FLAURA demonstrated that osimertinib is indicated as the first-line treatment for metastatic patients with NSCLC having EGFR sensitizing mutations and for patients with metastatic NSCLC having EGFR T790M mutations whose disease has progressed or after the treatment with first- or second-generation EGFR TKI (5, 6). However, notable toxicities related to EGFR TKIs includes acneiform rash, diarrhea, paronychia, and interstitial lung disease (ILD)/pneumonitis which can lead to fatal outcomes. The incidence of ILD with the first- and second-generation EGFR-TKI has been reported to occur in 3–5% of East Asians, which is substantially higher than in Caucasians (7, 8). Osimertinib-induced ILD in Japanese patients has been reported to occur in 12–18% for any grades and 2–4.6% for grade 3 or higher (9, 10).

ICIs, such as cytotoxic T-lymphocyte antigen-4, programmed cell death protein-1 (PD-1), and its ligand PD-L1, target down-regulators of the anticancer immune response, unleashing the host immune reaction against tumor cells by T-cell activation. ICIs generate unique immune-mediated side-effects, called immune-related adverse events (irAEs), in almost all organs (11). ICI-induced ILD is a major concern, particularly in patients with NSCLC. Its incidence is 3–5%, regardless of race and may lead to fatal outcomes; therefore, careful attention is warranted (12–14).

Tumor angiogenesis is indispensable for tumor proliferation and metastasis. Abnormalities in the vascular endothelial growth factor/vascular endothelial growth factor receptor (VEGF/VEGFR) pathway have been recognized as key factors in tumor angiogenesis (15, 16). In the treatment of NSCLC, several monoclonal antibodies, such as bevacizumab and ramucirumab, and small-molecule inhibitors, such as vandetanib, sunitinib, nintedanib, and anlotinib, have been developed to interrupt the interaction between VEGF and its receptors in order to achieve a therapeutic effect (17, 18). Furthermore, VEGF and EGF share common downstream signaling pathways and may function exclusively with each other during tumor progression and acquired therapeutic resistance (19, 20). Therefore, the approaches toward dual blockade of these molecular targets have been developed to demonstrate promising results in the treatment of advanced NSCLC. Several randomized studies have demonstrated the combination therapy with EGFR and VEGF/VEGFR inhibitors has superior progression-free survival than that with EGFR inhibitors in patients with untreated EGFR-mutated metastatic NSCLC (21–24). In cancer immunotherapy, VEGF is not only an important angiogenic factor but also an immunomodulator of the tumor microenvironment (TME). VEGFs can suppress antigen presentation and stimulate the activity of regulatory T (Treg) cells, and tumor-associated macrophages, which in turn promote an immunosuppressive microenvironment in NSCLC (25–27). Therefore, blockade of the VEGF/VEGFR pathway may overcome therapeutic resistance to ICIs. Several randomized studies have demonstrated that combination therapy with ICIs and VEGF/VEGFR inhibitors resulted in superior progression-free survival than that with ICIs alone in patients with metastatic NSCLC (28, 29).

Combination therapy with VEGF/VEGFR inhibitors generally causes adverse events more frequently, and of higher grade, than monotherapy. However, some randomized studies have demonstrated a lower incidence and grade of ILD caused by EGFR TKI in combination therapy with VEGF/VEGFR inhibitors than in monotherapy, although the underlying reasons are still unknown (24, 30). Since the key endpoints in these studies were the efficacy of progression-free survival and overall survival, statistical power of the incidence of ILD was low. In this review, we reported that the addition of VEGF/VEGFR inhibitors can reduce the incidence of drug-induced ILD caused by EGFR TKI or ICI in patients with NSCLC.

## Methods

This systematic review followed the Preferred Reporting Items for Systematic Reviews and Meta-analyses (PRISMA) reporting guidelines (31) and was prospectively registered at PROSPERO (CRD42023409534).

### Primary and secondary objectives

The primary objective was to understand the odds ratio (OR) of the incidence of ILD induced by EGFR-TKIs and ICIs in all patients worldwide and Asians. Secondary objectives to explore the odds ratios of the incidence of ILD at grade 3 or higher induced by EGFR-TKIs and ICIs in all patients worldwide and Asians.

### Eligibility criteria

Potentially eligible studies had to meet the following criteria: 1) randomized phase 2 or 3 studies in advanced or metastatic (stage IIIB, IIIC, IV, and postoperative recurrence) NSCLC treated with either EGFR-TKI or ICI; 2) studies published in English from January 1st, 2009, to October 31st, 2023; 3) availability of data on the incidence and grade of drug-induced ILD such as pneumonitis, pneumonia, and acute lung injury (The data on lung infection and respiratory tract infection were excluded from drug-induced ILD); 4-a) In EGFR-TKI group, the control arm was an EGFR TKI (± placebo) and the experimental (VEGF/VEGFR) arm was EGFR TKI plus VEGF/VEGFR inhibitors; 4-b) In ICI group, the control arm was an ICI ± cytotoxic chemotherapy (± placebo) and the experimental (VEGF/VEGFR) arm was an ICI ± cytotoxic chemotherapy plus VEGF/VEGFR inhibitors.

Studies were excluded if they met the following criteria: 1) studies of combination therapy with EGFR-TKIs and ICIs; and 2) retrospective studies, reviews, case reports, meta-analyses, or non-English publications.

Institutional review board approval and written informed consent are not required for database analysis.

### Search strategy

A systematic review was conducted using medical subject heading terms and text words related to EGFR inhibitors, ICI, and VEGF inhibitors in lung cancer while searching for relevant publications or presentations between January 2009 and October 2023 across PubMed, EMBASE, and the Cochrane Controlled Trials Register. Additionally, we analyzed abstracts and presentation contents from the meetings of the American Society of Clinical Oncology (ASCO), the European Society of Medical Oncology (ESMO), the World Conference on Lung Cancer (WCLC), and other pivotal annual meetings for lung cancer.

The search terms are listed in **Supplementary Table S1**. Publications and abstracts were extracted based on the eligibility criteria, and the data were reviewed thereafter for discrepancies and inconsistencies. In case of duplicate publications, the most recent version of the publication was included, with older publications referred to only if the primary or secondary outcomes were not reported in the most recent study.

### Data collection process

From all the included studies, we collected the basic information, including the authors’ names, date of publication, name of publication journal or meeting, phase of the study, therapeutic regimen, treatment line, subject size (number), participants’ characteristics (race, age, sex, smoking status, ECOG-PS, EGFR mutation type, PD-L1 TPS, etc.), and the incidence and grade of ILD/pneumonitis.

### Quality analysis

A risk-of-bias table was generated for the included trials using the Cochrane risk-of-bias domains of random sequence generation, allocation concealment, blinding of participants/physicians, blinding of outcome assessment, incomplete outcome data, and selective reporting (32). Quality of the method, the risk of bias in the study, and the discrepancies and inconsistencies were independently reviewed in this study.

### Statistical analysis

We calculated the odds ratios (ORs) and 95% confidence intervals (CIs) for ILD from all studies and synthesized the data. We used a random-effects model to perform the meta-analysis. Heterogeneity among studies was assessed using chi-square-based Q statistics and I² statistics. We used funnel plot to investigate potential publication bias, when there were 10 or more research articles. All statistical analyses were performed using Review Manager Software, version 5.4 (The Cochrane Collaboration, Copenhagen: The Nordic Cochrane Centre, 2020). A P value of < 0.05 indicated statistically significant difference.

## Results

### Study selection

**Figure 1:**
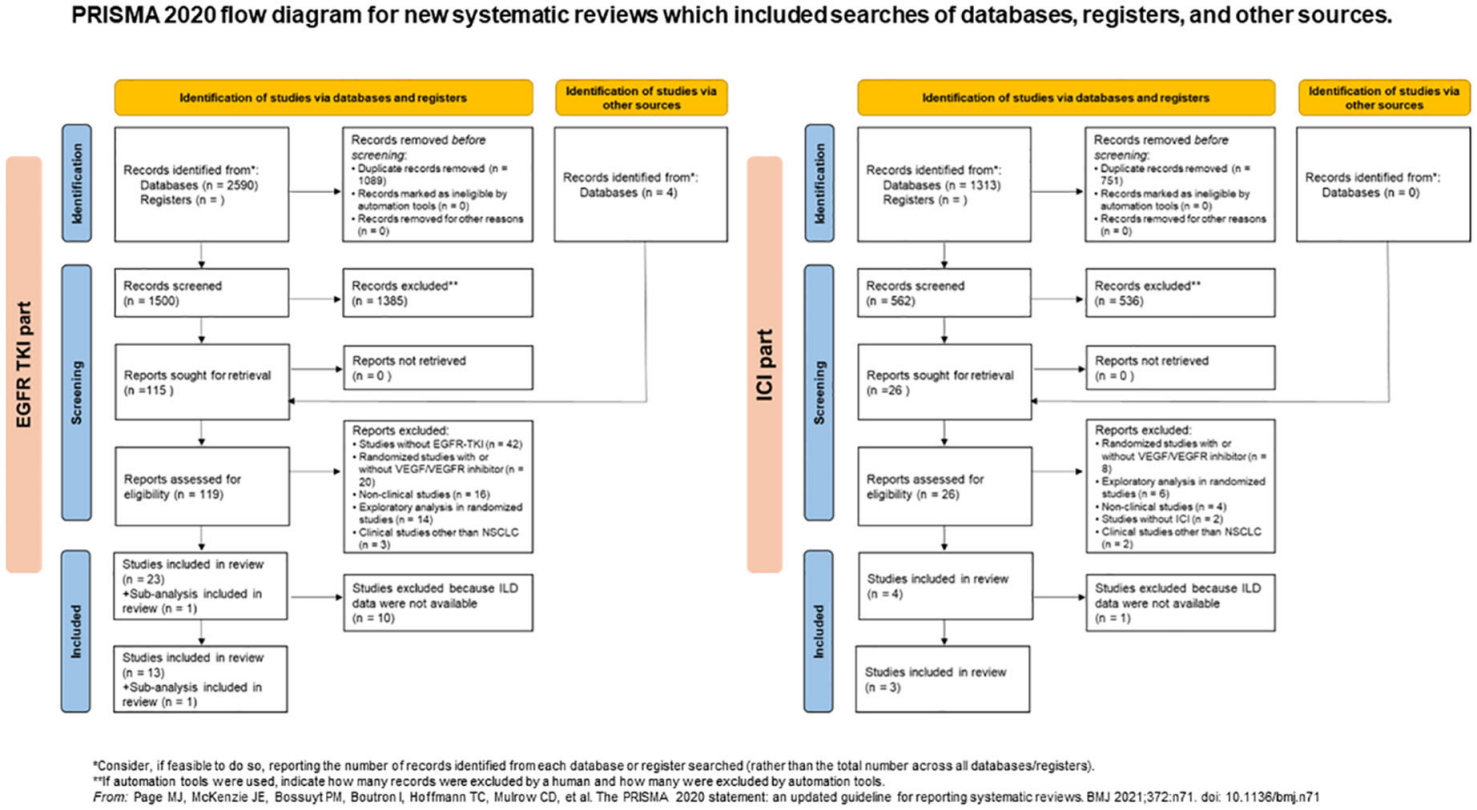
PRISMA 2020 flow diagram for new systematic reviews which included searches of databases, registers, and other sources. EGFR, epidermal growth factor receptor; ICI, immune checkpoint inhibitor, interstitial lung disease; ILD, interstitial lung disease; NSCLC, non-small lung cancer; TKI, tyrosine kinase inhibitor.

Our search extracted 119 publications in the EGFR-TKI group and 26 publications in the ICI group between January 2009 and October 2023. After removing duplicates and studies that did not meet the inclusion criteria, we identified 23 randomized studies in the EGFR-TKI group and four randomized studies in the ICI group, based on the eligibility criteria. In a randomized phase III RELAY study of ramucirumab plus erlotinib in patients with untreated, EGFR-mutated, advanced NSCLC, the results were published after primary analysis, performed separately in two sub-analyses for the East Asian and Europe/United States subsets (23, 33, 34). A total of 29 publications including these sub-analyses, are listed in **Table 1**. Thirteen studies and one sub-analysis in the EGFR-TKI group and three in the ICI group were extracted, owing to the selection of studies for which ILD data were available in the publications (**Figure 1**). Among the studies, BeTa study did not include ILD data for any grade, except for grade ≥ 3 (21). Therefore, we performed a meta-analysis of the EGFR-TKI group to evaluate the OR of ILD incidence for any grade across 12 studies and one sub-analysis, and to evaluate the OR of ILD incidence for grade ≥ 3 in 13 studies and one sub-analysis.

**Table 1:** Summary of all randomized trials included in the systematic review.

Author	Year	Study name Digital object identifier	Phase	Cancer type	Treatment line	Race	Treatment regimen in control arm	VEGFi	EGFR-TKI with VEGFi				EGFR-TKI without VEGFi				Odds ratio	
EGFR TKI part									N	Any grade	Grade 1–2	Grade ≥3	N	Any grade	Grade 1–2	Grade ≥3	Any grade	Grade ≥3
Spigel DR	2011	LUN160 doi: 10.1200/JCO.2010.30.7678 (35).	Phase 2	NSCLC	2^nd^ or 3^rd^	White 86.7% African American 12.7%	Erlotinib	Sorafenib	111	NA	NA	NA	55	NA	NA	NA	–	–
Herbst RS	2011	BeTa doi: 10.1016/S0140–6736 (11)60545-X (21).	Phase 3	NSCLC	2^nd^	White 81.9% Black 8.5% Asian 6.4%	Erlotinib	Bevacizumab	313	NA	NA	2	313	NA	NA	2	–	1.00
Scagliotti GV	2012	NCT00457392 doi: 10.1200/JCO.2011.39.2993 (36).	Phase 3	NSCLC	2^nd^ or later	White 85.9% Asian 10.7% Black plus other 3.3%	Erlotinib	Sunitinib	480	NA	NA	NA	480	NA	NA	NA	–	–
Groen HJM	2013	NCT00265317 doi: 10.1093/annonc/mdt212 (37).	Phase 2	NSCLC	2^nd^	Caucasian 96.2% Asian 2.3%	Erlotinib	Sunitinib	64	NA	NA	NA	64	NA	NA	NA	–	–
Neal JW	2016	ECOG-ACRIN 1512 doi: 10.1016/S1470–2045 (16)30561–7 (38).	Phase 2	NSCLC	2^nd^ or 3^rd^	White 74.1% Asian 2.3%	Erlotinib	Cabozantinib	39	1 (2.56%)	0	1 (2.56%)	40	1 (2.50%)	1 (2.50%)	0	1.026	–
Wang Y	2017	doi: 10.1016/j.biopha.2017.02.097 (39).	Phase 3	NSCLC EGFR mut+ 42.1%, EGFR wild 35.0%, Unkown 22.9%	2^nd^	Chinese	Erlotinib	Bevacizumab and panitumumab	150	NA	NA	NA	147	NA	NA	NA	–	–
Kato T	2018	JO25567: Update safety results doi: 10.1007/s40264–017-0596–0 (40).	Phase 2	EGFR-mutated NSCLC	1^st^	Japanese	Erlotinib	Bevacizumab	75	2 (2.67%)	2 (2.67%)	0	77	3 (3.90%)	3 (3.90%)	0	0.676	0
Spigel DR	2018	doi: 10.1002/cncr.31290 (41).	Phase 2	NSCLC	2^nd^ or 3^rd^	American	Erlotinib	Pazopanib	126	NA	NA	NA	64	NA	NA	NA	–	–
Kitagawa C	2019	UMIN000013586 doi: 10.21873/invivo.11498 (42).	Phase 2	EGFR**-**mutated NSCLC	1^st^	Japanese	Gefitinib	Bevacizumab	6	1 (16.67%)	1 (16.67%)	0	10	0	0	0	–	–
Saito H	2019	NEJ026 doi: 10.1016/S1470–2045 (19)30035-X (24).	Phase 3	EGFR**-**mutated NSCLC	1^st^	Japanese	Erlotinib	Bevacizumab	112	0	0	0	114	5	5 (4.39%)	0	–	–
Stinchcombe TE	2019	NCT01532089 doi: 10.1001/jamaoncol.2019.1847 (43).	Phase 2	EGFR-mutated NSCLC	1^st^	White 85.2%, Asian 3.4%	Erlotinib	Bevacizumab	43	0 NA	NA	NA	45	0 NA	NA	NA		
Nakagawa K	2019	RELAY study doi: 10.1016/S1470–2045 (19)30634–5 (23).	Phase 3	EGFR-mutated NSCLC	1^st^	Asian 77% White 22.2% Other 0.6%	Erlotinib	Ramucirumab	221	4 (1.80%)	3 (1.36%)	1 (0.45%)	225	6 (2.67%)	4 (1.78%)	2 (0.89%)	0.673	0.507
Nishio M	2020	RELAY study East Asian subset doi: 10.1111/cas.14655 (33).	Phase 3	EGFR**-**mutated NSCLC	1^st^	Asian	Erlotinib	Ramucirumab	164	3 (1.83%)	2 (1.22%)	1 (0.61%)	170	6 (3.53%)	3 (1.76%)	3 (1.76%)	0.509	0.342
Aix SP	2021	RELAY study Europe/United States subset doi: 10.1016/j.ctarc.2021.100378 (34).	Phase 3	EGFR**-**mutated NSCLC	1^st^	Caucasian 88.5% Asian 8.9%	Erlotinib	Ramucirumab	57	1 (1.75%)	1 (1.75%)	0	55	0	0	0	–	–
Akamatsu H	2021	WJOG8715L doi: 10.1001/jamaoncol.2020.6758 (44).	Phase 2	EGFR T790M-mutated NSCLC	2^nd^ or later	Japanese	Osimertinib	Bevacizumab	40	4 (10.00%)	4 (10.00%)	0	41	5 (12.20%)	5 (12.20%)	0	0.80	
Zhao H	2021	ACTIVE study (CTONG1706) doi: 10.1016/j.jtho.2021.05.006 (45).	Phase 3	EGFR-mutated NSCLC	1^st^	Chinese	Gefitinib	Apatinib	157	1 (0.64%)	0	1 (0.64%)	154	2 (1.30%)	1 (0.65%)	1 (0.65%)	0.487	0.981
Zhou Q	2021	ARTEMIS-CTONG1509 doi: 10.1016/j.ccell.2021.07.005 (46).	Phase 3	EGFR**-**mutated NSCLC	1^st^	Chinese	Erlotinib	Bevacizumab	157	1 (0.64%)	0	1 (0.64%)	153	1 (0.65%)	1 (0.65%)	0	0.974	
Soo RA	2022	ETOP 10–16 BOOSTER trial doi: 10.1016/j.annonc.2021.11.010 (47).	Phase 2	EGFR T790M-mutated NSCLC	2^nd^	Asian 41.0% Non-Asian 59.0%	Osimertinib	Bevacizumab	76	NA	NA	NA	77	NA	NA	NA		
Kenmotsu H	2022	WJOG9717L doi: 10.1016/j.jtho.2022.05.006 (30).	Phase 2	EGFR**-**mutated NSCLC	1^st^	Japanese	Osimertinib	Bevacizumab	61	2 (3.28%)	1 (1.64%)	1 (1.64%)	60	11 (18.33%)	10 (16.67%)	1 (1.67%)	0.151	0.983
Piccirill MC	2022	BEVERLY doi: 10.1016/j.jtho.2022.05.008 (48).	Phase 3	EGFR**-**mutated NSCLC	1^st^	Italian	Erlotinib	Bevacizumab	80	0	NA	NA	79	0	NA	NA		
Ninomiya T	2023	AfaBev-CS doi: 10.1016/j.lungcan.2023.107349 (49).	Phase 2	EGFR**-**mutated NSCLC	1^st^	Japanese	Afatinib	Bevacizumab	49	1 (2.04%)	1 (2.04%)	0	50	3 (6.00%)	2 (4.00%)	1 (2.00%)	0.326	NA
Lee Y	2023	NCT03126799 doi: 10.1002/cncr.34553 (50).	Phase 2	EGFR**-**mutated NSCLC +	1^st^	Korean	Erlotinib	Bevacizumab	64	2 (3.13%)	0	2 (3.13%)	63	2 (3.17%)	1 (1.59%)	1 (1.59%)	0.984	2.00
Nakahara Y	2023	OSIRAM-1 doi: 10.1016/j.annonc.2023.10.071 (51).	Phase 2	EGFR**-**mutated NSCLC +	1^st^	Japanese	Osimertinib	Ramucirumab	59	5 (8.5%)	4 (6.7%)	1 (1.7%)	62	10 (16.1%)	9 (14.5%)	1 (1.6%)	0.481	1.05
Wang J	2023	NCT04425187 doi: 10.1016/j.annonc.2023.09.2365 (52).	Phase 2	EGFR L858R-mutated NSCLC +	1^st^	Chinese	Gefitinib	Bevacizumab	41	NA	NA	NA	39	NA	NA	NA		
Le X	2023	RAMOSE doi: 10.1016/j.annonc.2023.10.072 (53).	Phase 2	EGFR**-**mutated NSCLC +	1^st^	White 64.9%, Asian 24.5%, Black 3.6%	Osimertinib	Ramucirumab	93	NA	NA	NA	46	NA	NA	NA		
ICI part									N	Any grade	Grade 1–2	Grade ≥ 3	N	Any grade	Grade 1–2	Grade ≥ 3	Any grade	Grade ≥ 3
Reck M	2020	IMpower 150 doi: 10.1200/JCO.19.03158 (54).	Phase 3	Lung adenocarcinoma	1^st^	White 82.1% Asian 12.8% Black 1.9% other 3.3%	Atezolizumab, carboplatin, and paclitaxel	Bevacizumab	393	13 (3.31%)	7 (1.78%)	6 (1.53%)	400	23 (5.75%)	13 (3.25%)	10 (2.50%)	0.561	0.605
Shiraishi Y	2023	WJOG11218L (APPLE study) doi: 10.1001/jamaoncol.2023.5258 (55).	Phase 3	Lung adenocarcinoma	1^st^	Japanese	Atezolizumab, carboplatin, and pemetrexed	Bevacizumab	205	21 (10.24%)	12 (5.85%)	9 (4.39%)	205	19 (9.27%)	13 (6.34%)	6 (2.93%)	1.117	1.523
Lu S	2023	ORIENT-31 doi: 10.1016/S2213–2600 (23)00135–2 (29).	Phase 3	EGFR-mutated NSCLC	2^nd^	Chinese	Sintilimab plus chemotherapy	IBI305, which is bevacizumab-biosimilar	158	8 (5.06%)	7 (4.43%)	1 (0.63%)	156	11 (7.05%)	5 (3.21%)	6 (3.85%)	0.703	0.159
Zhang W	2023	NCT03910127 Doi: 10.1016/j.lungcan.2023.107353 (56).	Phase 2	Driver-negative NSCLC	2^nd^ or later	Chinese	TQB2450 (PD-L1)	Anlotinib (10 or 12 mg)	68	NA	NA	NA	33	NA	NA	NA	NA	NA

NA means "not available".

"-" means blank data, because value is not calculated.

### Study characteristics

The breakdown of EGFR-TKIs was as follows: erlotinib in seven of 13 studies, osimertinib in three studies, gefitinib in two studies, afatinib in one study, VEGF/VEGFR inhibitors in nine studies, ramucirumab in two studies, and VEGF TKIs in two studies. In the ICI group, the ICIs in combination with cytotoxic chemotherapy were atezolizumab in two studies and sintilimab in one study whereas all VEGF/VEGFR inhibitors were bevacizumab or its biosimilar antibody.

Thirteen studies in the EGFR-TKI group included 2,715 patients (1,353 in the VEGF/VEGFR arm and 1,362 in the control arm), with individual study sizes varying from 16 to 626 patients. Three studies in the ICI group included 1,517 patients (756 in the VEGF/VEGFR arm and 761 in the control arm), with individual study sizes ranging from 314 to 793 patients. Among the 13 studies in the EGFR-TKI group, ten studies and one sub-analysis were conducted only in Asian countries (7 in Japan, 2 in China, one in Korea, and one in an East Asian country). Eleven Asian studies, along with one sub-analysis, included 1,898 patients (944 in the VEGF/VEGFR arm and 954 in the control arm), with individual study sizes varying from 16 to 334 patients. Among the three studies in the ICI group, two were conducted in Asian countries (one each in China and Japan). The studies included 724 patients (363 and 361 in the VEGF/VEGFR and control arms, respectively).

### Risk of bias in studies

**Figure 2:**
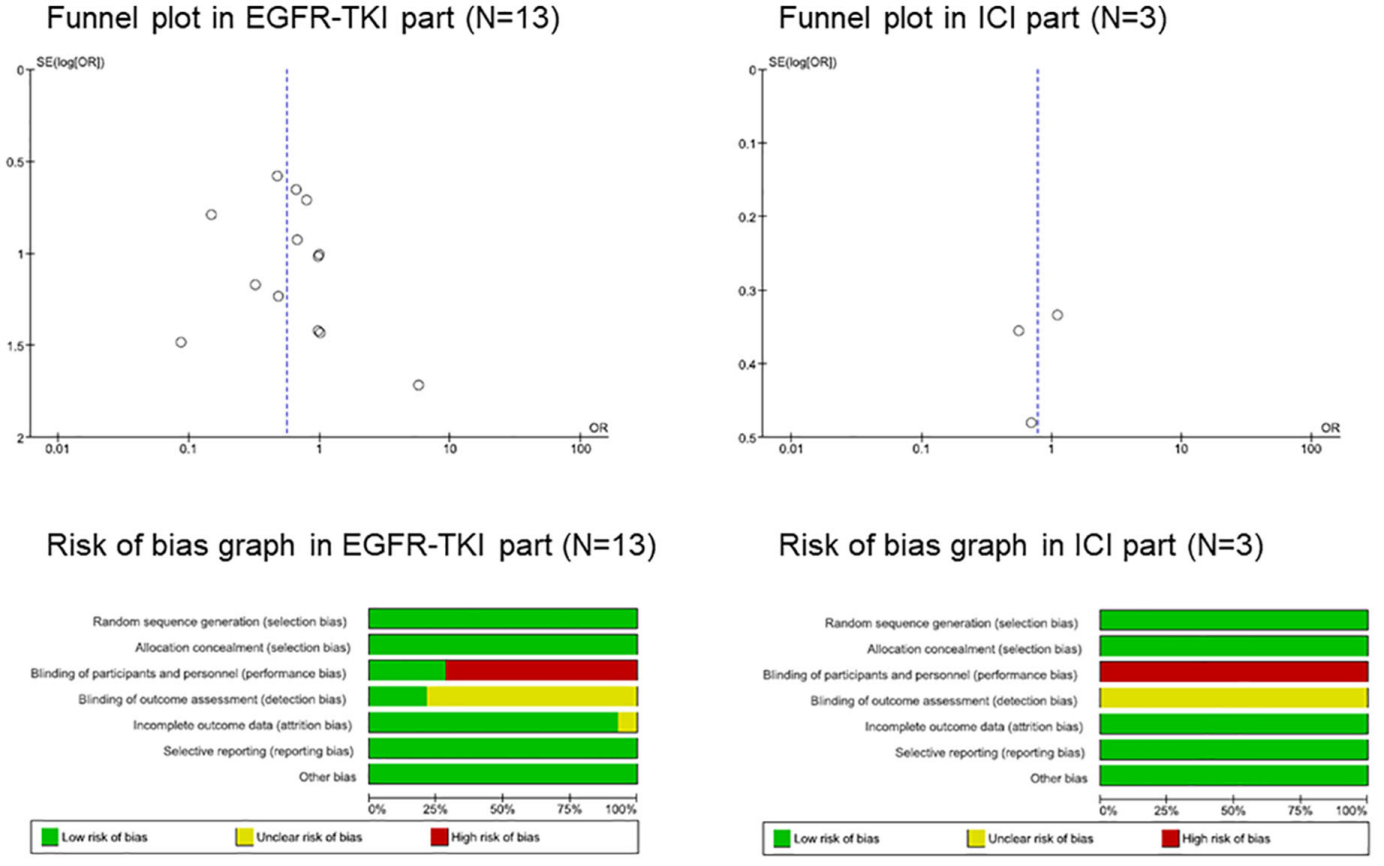
Funnel plot and risk of bias graph. EGFR, epidermal growth factor receptor; ICI, immune checkpoint inhibitor, interstitial lung disease; TKI, tyrosine kinase inhibitor.

Since the 13 studies were extracted from the EGFR-TKI group, funnel plots were used to evaluate the publication bias. However, since only three studies were extracted from the ICI group, further analyses were performed to evaluate the potential risk. Visual inspection of the funnel plots for the ORs in the EGFR-TKI group revealed no asymmetry (**Figure 2**). Our understanding about each risk-of-bias item is presented as a percentage across all included studies that showed a low risk of bias.

Randomized trials can be divided into double-blind, placebo-controlled, and open-label. We classified the blinding of participants and personnel (performance bias) as low-risk in double-blind placebo-controlled trials and as high-risk in open-label trials, and the blinding of outcome assessment (detection bias) as low-risk in double-blind placebo-controlled trials and uncertainty risk in open-label trials (**Figure 2**; **Supplementary Figure S1**).

### Results of individual studies

**Figure 3:**
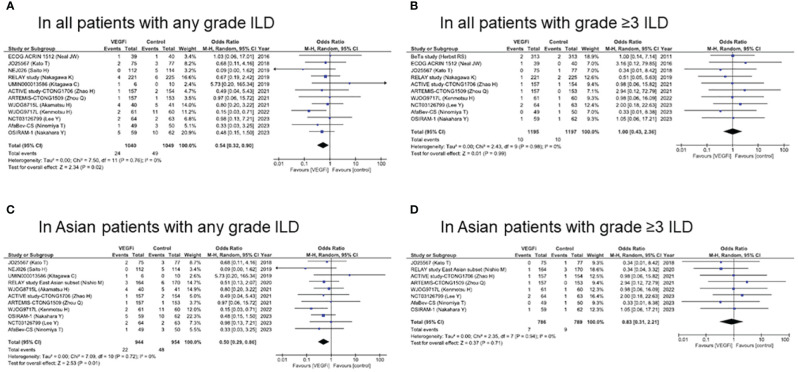
Forest plot and pooled odds ratio of ILD caused by EGFR-TKI in all patients **(A, B)** and in Asian patients **(C, D)** with/without VEGF/VEGFR inhibitors. EGFR, epidermal growth factor receptor; ILD, interstitial lung disease; TKI, tyrosine kinase inhibitor; VEGF, vascular endothelial growth factor; VEGFR, vascular endothelial growth factor receptor.

Meta-analysis was performed to evaluate the OR of ILD incidence with or without VEGF/VEGFR inhibitors using a random-effects model (**Figure 3A**). In all subjects in the EGFR-TKI group, the OR of ILD incidence at any grade with VEGF/VEGFR inhibitors was 0.54 (95% CI, 0.32–0.90; p = 0.02), which represented a significantly lower incidence than that without VEGF/VEGFR inhibitors. On the other hand, the OR of ILD incidence at grade ≥ 3 with VEGF/VEGFR inhibitors was 1.00 (95% CI, 0.43–2.36; p = 0.99), which did not represent a significant incidence compared to that without VEGF/VEGFR inhibitors (**Figure 3B**).

In Asian subjects in the EGFR-TKI group, the OR of ILD incidence at any grade with VEGF/VEGFR inhibitors was 0.50 (95% CI, 0.29–0.86; p = 0.01), which indicated a significantly lower incidence than that without VEGF/VEGFR inhibitors (**Figure 3C**). On the other hand, the OR of ILD incidence at grade ≥ 3 with VEGF/VEGFR inhibitors was 0.83 (95% CI, 0.31–2.21; p = 0.71) (**Figure 3D**). Forest plots by each EGFR-TKI and by each VEGF/VEGFR inhibitors were presented in **Supplementary Figures S2**, **S3**, respectively. In all subjects treated with erlotinib (n=7), the OR of ILD incidence at any grade with VEGF/VEGFR inhibitors was 0.70 (95% CI, 0.33–1.47; p = 0.34). In all subjects treated with EGFR-TKI, the OR of ILD incidence at any grade with bevacizumab (n=8) was 0.51 (95% CI, 0.23–1.03; p = 0.06).

In all subjects in the ICI group, the OR of ILD incidence at any grade with VEGF/VEGFR inhibitors was 0.78 (95% CI, 0.51–1.21; p = 0.27) and that at grade ≥ 3 with VEGF/VEGFR inhibitors was 0.69 (95% CI, 0.24–1.98; p = 0.49), which was not a significant incidence (**Supplementary Figure S4**). Additionally, in all subjects with advanced or metastatic NSCLC treated with either EGFR-TKI or ICI, the OR of ILD incidence at any grade with VEGF/VEGFR inhibitors was 0.67 (95% CI, 0.49–0.94; p = 0.02), which showed a significantly lower incidence than that without VEGF/VEGFR inhibitors. On the other hand, the OR of ILD incidence at grade ≥ 3 with VEGF/VEGFR inhibitors was 0.87 (95% CI, 0.51–1.48; p = 0.60), which did not indicate a significant incidence.

## Discussion

This systematic review reported that the addition of VEGF/VEGFR inhibitors could reduce the incidence of drug-induced ILD at any grade caused by EGFR-TKI in patients with NSCLC, but not of those at grade ≥ 3, although odds ratio varied across clinical studies. Although the tendencies were maintained in an additional integrated analysis of drug-induced ILD caused by either EGFR-TKI or ICI, whether the incidence of drug-induced ILD caused by ICI could be reduced by VEGF/VEGFR inhibitors is yet to be determined due to the limited number of randomized trials with ILD data available.

A previous systematic review of the efficacy and toxicity in patients with EGFR-mutated NSCLC had shown that combined inhibition of the EGFR and VEGF pathways significantly increased any grade 3–4 toxicity compared to EGFR inhibition alone (57). However, the current systematic review did not refer to drug-induced ILD/pneumonitis at grade 1–2 toxicities. Another systematic review had investigated whether the addition of bevacizumab could reduce the incidence of drug-induced ILD at any grade caused by cancer drug therapies, including cytotoxic agents, antibodies, TKI, and ICI in patients with malignant solid tumors. The systematic review demonstrated that the odds ratio for ILD in the bevacizumab group was 0.62 (95% CI 0.42–0.92; p = 0.02), which showed a significantly lower incidence than in the control. This tendency was observed in the targeted therapy groups but not in the cytotoxic agent groups. The three systematic reviews mentioned above, including ours, consisted of many overlapping clinical trials identified by their respective database searches. However, the clinical questions addressed in each systematic review differed. ILD induced by anti-cancer agents is known to occur more frequently in lung cancer than in other solid tumors (23, 58–62). Therefore, we concluded that the addition of VEGF/VEGFR inhibitors could reduce the incidence of drug-induced ILD caused by EGFR-TKI or ICI in patients with NSCLC.

VEGF is considered to play an important role in pathogenesis of acute exacerbation of interstitial pulmonary fibrosis (63). In preclinical model, VEGF expression is associated with angiogenesis and positive remodeling of damaged tissues, which increase vascular permeability and pulmonary edema, resulting in acute lung injury (64). The VEGF inhibitor CBO-P11 suppressed the expression of key mediators of pro- and antifibrotic responses in a bleomycin-induced pulmonary fibrosis model (65). On the other hand, an imbalance in VEGF splice isoforms has been reported to be important in the development of pulmonary fibrosis (66). Clinical studies has shown that nintedanib, a multi-targeted TKI of VEGF, PDGF, and FGF, is effective in reducing the decline of forced vital capacity in patients with progressive fibrosing ILD and decreasing the events of ILD progression (67–69). However, in the J-SONIC study for advanced NSCLC with idiopathic pulmonary fibrosis, nintedanib plus chemotherapy did not improve the exacerbation-free survival compared with chemotherapy alone (HR 0.89, 90% CI 0.67–1.17; p=0.24) (70). The mechanisms by which VEGF inhibitors reduce the incidence of drug-induced ILD remains to be elucidated.

The prognosis of NSCLC has improved dramatically with the advent of molecular-targeted agents for patients with driver gene alterations and with the use of immune checkpoint inhibitors for patients without driver gene alterations. However, since severe adverse events can lead to fatal outcomes, drug-induced ILD or pneumonitis is a major concern in cancer treatment. In the clinical management of patients receiving molecular targeting agents and ICIs, the diagnosis of drug-induced ILD is performed using high-resolution CT and is usually achieved by excluding other potential known causes, such as infection or disease progression of primary cancer (71). When drug-induced ILD develops, the severity is assessed according to CTCAE, and careful monitoring and treatment, including corticosteroids and other immunosuppressive therapies, is initiated along with supportive measures, including supplemental oxygen and intensive care, based on its severity, suspected agent, and risk factors. The incidence of EGFR-TKI-induced ILD is genetically different between East Asians and Caucasians (7, 72–74). Therefore, drug-induced ILD is a major concern, particularly in East Asian patients with NSCLC.

Our current systematic review had a few limitations. First, the primary endpoint in almost all randomized trials was PFS or OS, not an adverse event, including drug-induced ILD. We analyzed 75 ILD events in the EGFR-TKI group and 94 ILD events in the ICI group included in our systematic review, which in general does not represent a large number of events. Second, the clinical characteristics were not the same in all comparison groups. Our systematic review included a variety of NSCLC types, including EGFR-mutated and wild-type, and treatment with EGFR inhibitors, ICI, and VEGF inhibitors. The incidence of ILD with the first- and second-generation EGFR-TKI has been reported to occur in 3–5% of East Asians and the incidence of ILD with osimertinib in 12–18% of East Asians (7–10). Such heterogeneity could lead to the possibility of bias from one trial to another. It is the limitation in this systematic review. Third, there are many differences between East Asian and Western countries in the reporting criteria for ILD and in the concerns among medical professionals regarding drug-induced ILD. In the phase 3 PACIFIC study of durvalumab in patients with locally advanced NSCLC after concurrent chemoradiotherapy, the incidence of any-grade pneumonitis was 33.9% in all patients and 73.6% in the Japanese subgroup, although the incidence of pneumonitis at grade ≥ 3 was 3.4% in all patients and 5.6% in the Japanese subgroup (75). In Japan, ILDs at grade 1 may have been reported more frequently owing to concerns about adverse effects. However, drug-induced ILD has not yet been assessed in many Western countries.

In conclusion, this systematic review demonstrated that the addition of VEGF/VEGFR inhibitors could reduce the incidence of drug-induced ILD at any grade caused by EGFR-TKI in patients with NSCLC, but not in grade ≥ 3. In ICI-induced ILD, whether the incidence could be reduced remains to be determined owing to the limited number of randomized trials for which ILD data are available.

## Data availability statement

The raw data supporting the conclusions of this article will be made available by the authors, without undue reservation.

## Author contributions

YF: Conceptualization, Data curation, Formal analysis, Investigation, Methodology, Project administration, Resources, Validation, Visualization, Writing – original draft, Writing – review & editing. KS: Data curation, Formal analysis, Methodology, Supervision, Validation, Visualization, Writing – original draft, Writing – review & editing. TY: Conceptualization, Methodology, Writing – review & editing. JS: Writing – review & editing. NW: Writing – review & editing. RM: Writing – review & editing. KM: Formal analysis, Methodology, Supervision, Validation, Writing – review & editing. YH: Writing – review & editing.

## Supplementary material

Supplementary Table 1 Literature search strategy. EGFR, epidermal growth factor receptor; ICI, immune checkpoint inhibitor, interstitial lung disease; TKI, tyrosine kinase inhibitor.

Supplementary Figure 2 Forest plot and pooled odds ratio of ILD by EGFR-TKI with/without VEGF/VEGFR inhibitors. **(A)** erlotinib, **(B)** osimertinib, **(C)** gefitinib, and **(D)** afatinib.

Supplementary Figure 3 Forest plot and pooled odds ratio of ILD by EGFR-TKI with/without VEGF/VEGFR inhibitors. **(A)** bevacizumab and **(B)** ramucirumab.
