# Supplementary material for: The incidence of drug-induced interstitial lung disease caused by epidermal growth factor receptor tyrosine kinase inhibitors or immune checkpoint inhibitors in patients with non-small cell lung cancer in presence and absence of vascular endothelial growth factor inhibitors: a systematic review
Source: Front Oncol. 2024 Jun 11;14:1419256. doi: 10.3389/fonc.2024.1419256 (PMC11196607; doi:10.3389/fonc.2024.1419256)
Supplement: Supplementary Table 1 — Literature search strategy. EGFR, epidermal growth factor receptor; ICI, immune checkpoint inhibitor, interstitial lung disease; TKI, tyrosine kinase inhibitor. [file Table_1.docx]

Supplemental Table S1. Literature Search Strategy

1) EGFR-TKI part

(“lung neoplasms”[Majr] OR “carcinoma, non-small-cell lung” OR “non-small cell lung cancer” OR “NSCLC” OR “advanced NSCLC” OR “metastatic NSCLC”) AND (“EGFR” OR “epidermal growth factor receptor” OR “EGFR-TKI” OR “epidermal growth factor receptor tyrosine kinase inhibitors” OR “tyrosine kinase inhibitor” OR “erlotinib” OR “gefitinib” OR “osimertinib” OR “dacomitinib” OR “afatinib” OR “icotinib” OR “aumolertinib” OR “lazertinib”) AND (“vascular endothelial growth factor” OR “vascular endothelial growth factor receptor inhibitors” OR “VEGF” OR “VEGF inhibitors” OR “VEGFR” OR “VEGFR inhibitors” OR “bevacizumab” OR “ramucirumab” OR “vandetanib” OR “sunitinib” OR “sorafenib” OR “nintedanib” OR “anlotinib” OR “apatinib” OR “IBI305”) AND "randomized controlled trial"[pt]

2) ICI part

(“lung neoplasms”[Majr] OR “carcinoma, non-small-cell lung” OR “non-small cell lung cancer” OR “NSCLC” OR “advanced NSCLC” OR “metastatic NSCLC”) AND (“Immune Checkpoint Inhibitors” OR “ICI” OR “Immune Checkpoint Blockers” OR “Immune Checkpoint Inhibition” OR “PD-1” OR “PD-L1” OR “CTLA-4” OR “PD-1 Inhibitors” OR “PD-L1 Inhibitor” OR “Programmed Death 1 Inhibitors” OR “Programmed Death Ligand 1 Inhibitors” OR “CTLA-4 Inhibitors” OR “Cytotoxic T-Lymphocyte-Associated Protein 4 Inhibitors” OR “atezolizumab” OR “avelumab” OR “cemiplimab” OR “durvalumab” OR “ipilimumab” OR “nivolumab” OR “pembrolizumab” OR “sintilimab” OR “tremelimumab”) AND (“vascular endothelial growth factor” OR “vascular endothelial growth factor receptor inhibitors” OR “VEGF” OR “VEGF inhibitors” OR “VEGFR” OR “VEGFR inhibitors” OR “bevacizumab” OR “ramucirumab” OR “vandetanib” OR “sunitinib” OR “sorafenib” OR “nintedanib” OR “anlotinib” OR “apatinib” OR “IBI305”) AND "randomized controlled trial"[pt]
