# Supplementary figures and images for: The incidence of drug-induced interstitial lung disease caused by epidermal growth factor receptor tyrosine kinase inhibitors or immune checkpoint inhibitors in patients with non-small cell lung cancer in presence and absence of vascular endothelial growth factor inhibitors: a systematic review
Source: Front Oncol. 2024 Jun 11;14:1419256. doi: 10.3389/fonc.2024.1419256 (PMC11196607; doi:10.3389/fonc.2024.1419256)

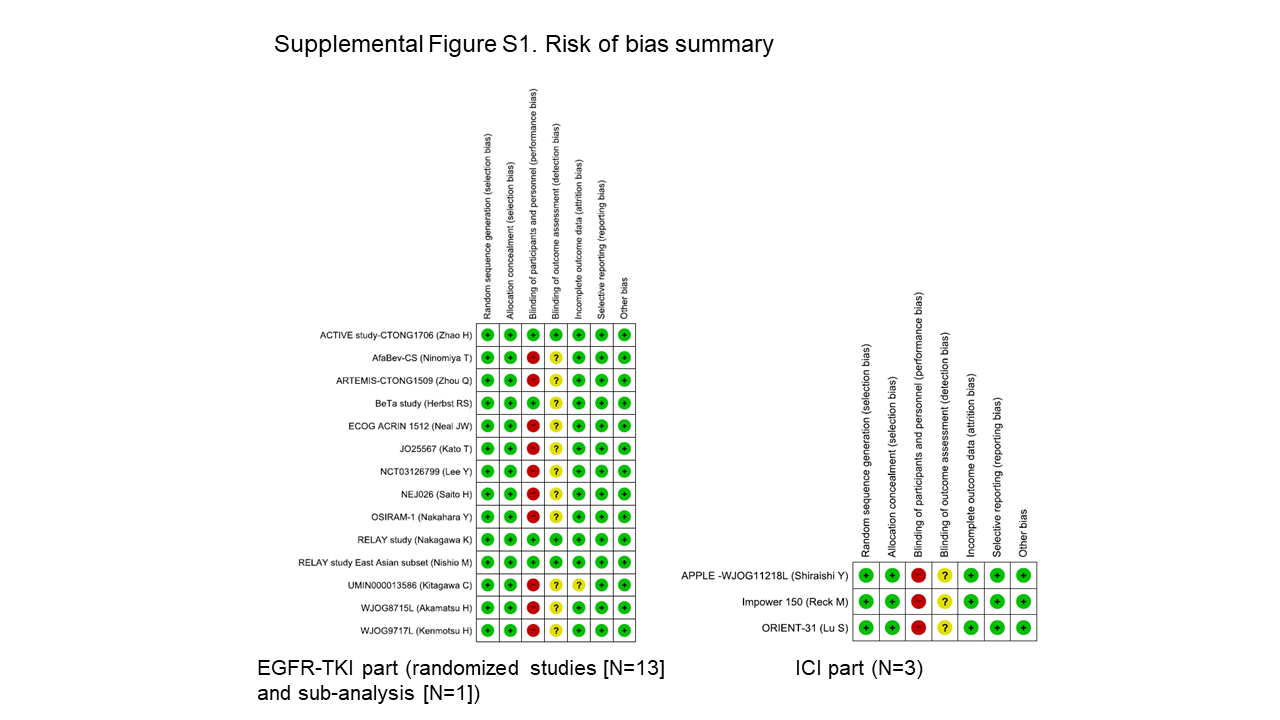

Supplement: Supplementary file 2 [file Image_1.tif]

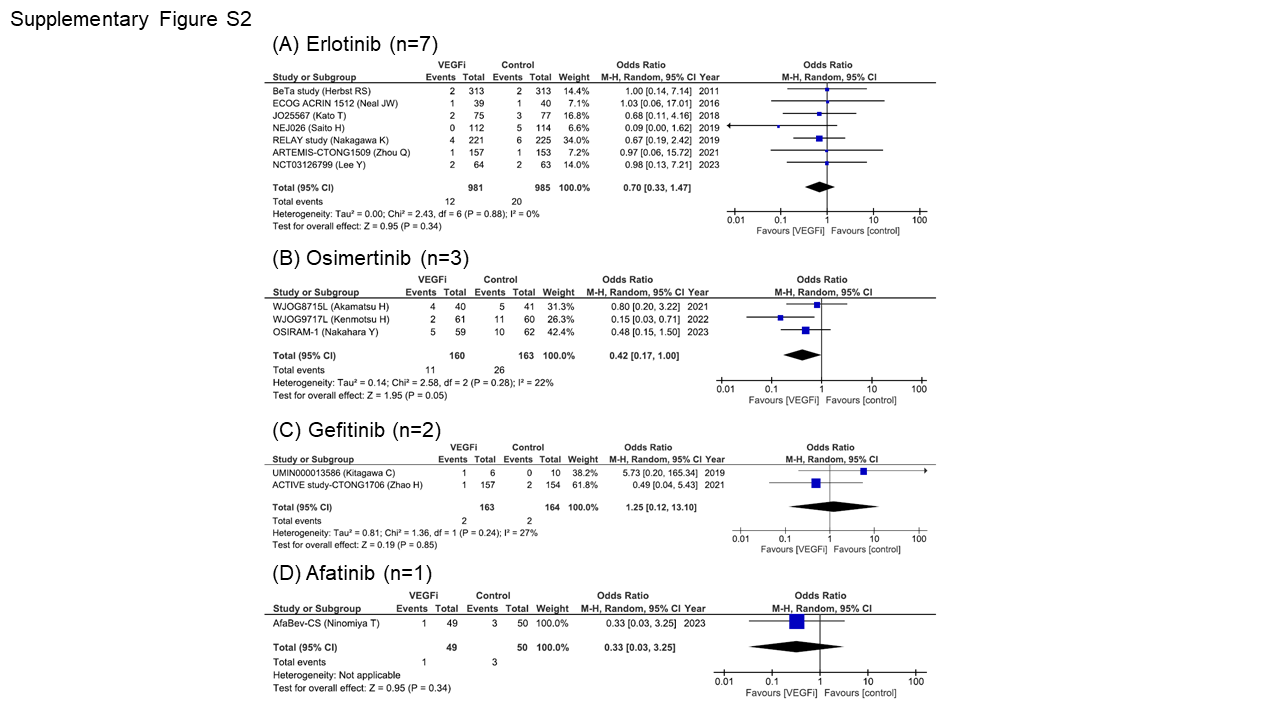

Supplement: Supplementary Figure 2 — Forest plot and pooled odds ratio of ILD by EGFR-TKI with/without VEGF/VEGFR inhibitors. (A) erlotinib, (B) osimertinib, (C) gefitinib, and (D) afatinib. [file Image_2.tif]

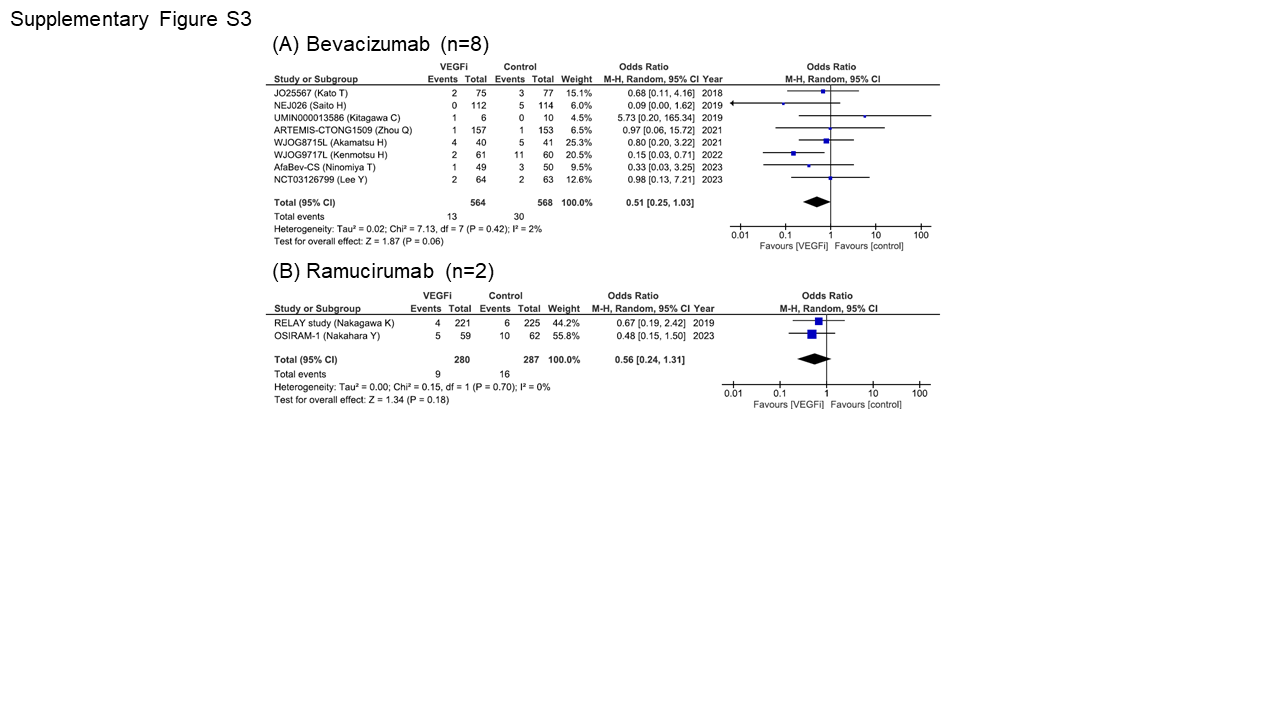

Supplement: Supplementary Figure 3 — Forest plot and pooled odds ratio of ILD by EGFR-TKI with/without VEGF/VEGFR inhibitors. (A) bevacizumab and (B) ramucirumab. [file Image_3.tif]

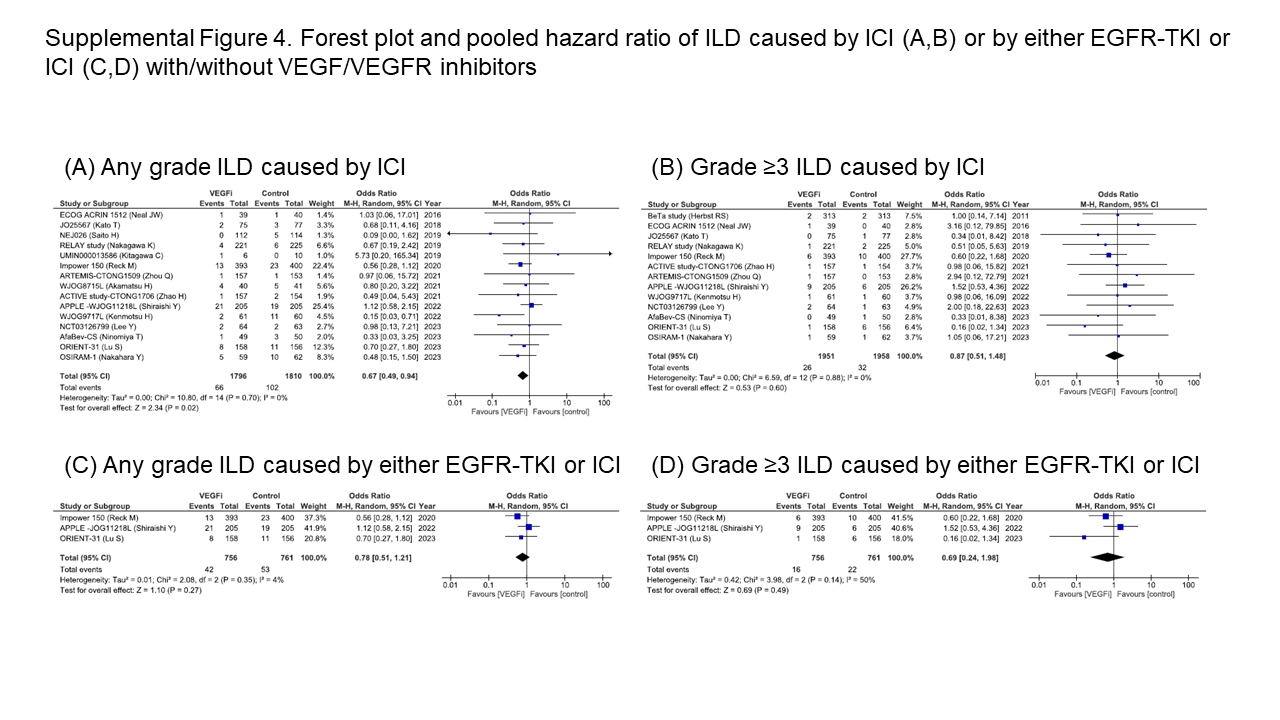

Supplement: Supplementary file 5 [file Image_4.tif]
